# A practical guideline for performing a comprehensive transthoracic echocardiogram in the congenital heart disease patient: consensus recommendations from the British Society of Echocardiography

**DOI:** 10.1186/s44156-022-00006-5

**Published:** 2022-10-18

**Authors:** Liam Corbett, Jan Forster, Wendy Gamlin, Nuno Duarte, Owen Burgess, Allan Harkness, Wei Li, John Simpson, Radwa Bedair

**Affiliations:** 1 Liverpool Heart and Chest Hospital NHS Foundation Trust, Liverpool, UK; 2 Leeds Teaching Hospital NHS Trust, Leeds, UK; 3 Manchester University NHS Foundation Trust, Manchester, UK; 4 University Hospitals Bristol and Weston NHS Foundation Trust, Bristol, UK; 5 East Suffolk and North Essex NHS Foundation Trust, Colchester, UK; 6 Royal Brompton and Harefield NHS Foundation Trust, Imperial College of London, London, UK; 7 Evelina London Children’s Hospital, Guy’s and St Thomas’ NHS Foundation Trust, London, UK

**Keywords:** Transthoracic echocardiography, Congenital heart disease, Sequential segmental analysis, Morphology

## Abstract

Transthoracic echocardiography is an essential tool in the diagnosis, assessment, and management of paediatric and adult populations with suspected or confirmed congenital heart disease. Congenital echocardiography is highly operator-dependent, requiring advanced technical acquisition and interpretative skill levels. This document is designed to complement previous congenital echocardiography literature by providing detailed practical echocardiography imaging guidance on sequential segmental analysis, and is intended for implementation predominantly, but not exclusively, within adult congenital heart disease settings. It encompasses the recommended dataset to be performed and is structured in the preferred order for a complete anatomical and functional sequential segmental congenital echocardiogram. It is recommended that this level of study be performed at least once on all patients being assessed by a specialist congenital cardiology service. This document will be supplemented by a series of practical pathology specific congenital echocardiography guidelines. Collectively, these will provide structure and standardisation to image acquisition and reporting, to ensure that all important information is collected and interpreted appropriately.

## Introduction

Advances in medical, surgical, and interventional congenital heart disease (CHD) management have led to marked improvements in paediatric CHD survival; with the number of adult congenital heart disease (ACHD) patients now exceeding that of their paediatric counterparts [1, 2]. Consequently, there is an increasing worldwide recognition for more specialised ACHD services. Life-long expert multi-modality imaging surveillance is of paramount importance for many CHD patients [3, 4], as they continue to experience CHD related cardiovascular sequalae [5]. Transthoracic echocardiography (TTE) has the ability to provide detailed information on cardiac morphology and haemodynamic physiology, and is largely accessible, non-invasive, and portable. Therefore, TTE remains the first line imaging tool in the diagnosis, assessment, and management of paediatric and adult populations with suspected or confirmed CHD [3–6].

Congenital TTE is a highly operator-dependent imaging practice that requires advanced technical acquisition and interpretative skill levels. Specialist training is required to ensure accurate assessment and reporting of cardiovascular malformations and outcomes of surgical/interventional procedures. Whilst thorough knowledge and understanding of cardiac morphology is central to paediatric congenital cardiology practice, it is somewhat a less familiar territory within adult cardiology practice [6]. Given most children with CHD now survive into adulthood, the ability to understand and perform congenital TTE in adult settings is of paramount importance. Standard adult TTE practices are largely designed to provide systematic guidance on performing a TTE study to detect and quantify acquired diseases in the structurally “normal” heart [7]. In CHD TTE, normal connections (i.e. cardiac morphology) cannot be assumed, and a knowledge of specific congenital lesions, their haemodynamic impact, and the surgical repair techniques is warranted. The British Society of Echocardiography (BSE) recognise that there is a need for more specialist CHD echocardiography practitioners and appreciate that there is limited opportunity to formally educate and practically train within CHD TTE nationwide. It is strongly recommended that any staff independently performing, and reporting CHD TTE studies are suitably qualified, i.e. BSE CHD, European Association for Cardiovascular Imaging (EACVI) CHD certified. This position will ensure all practitioners in CHD TTE are theoretically and practicably competent in understanding cardiac morphology, CHD nomenclature, the CHD pathology spectrum, and their associated repair/palliation methods. Importantly, this will enable practitioners to undertake a sequential segmental TTE approach to cardiac structure, avoiding interpretation based on assumed ‘normal’ cardiac morphology [6].

This document is designed to complement previous CHD TTE literature by providing detailed practical TTE imaging guidance on sequential segmental analysis [6–9], and is intended for implementation predominantly, but not exclusively, within ACHD settings. In contrast to a paediatric cardiology environment, anatomical diagnosis in ACHD settings is typically already established, aiding the TTE assessment. However, sometimes a patient’s historical TTE examination reflects very focused imaging. Therefore, it is recommended that a complete CHD TTE be performed at least once on all patients being assessed by a specialist congenital cardiology service, with subsequent CHD TTE studies being based on the patient’s anatomical and functional complexities, alongside patient amenability. It is therefore acceptable, within reason, that not all follow-up CHD TTE studies repeat a full sequential segmental anatomical evaluation, with the emphasis on pathology specific assessment.

This document will form the basis for a series of structured and practical pathology specific CHD TTE guidelines that will complement the BSE CHD accreditation curriculum. Collectively, these will help ensure important information is not missed or misinterpreted and aims to improve CHD TTE imaging quality by becoming an easy-to-use reference. It is anticipated that they will aid CHD TTE imaging and reporting standardisation, whilst also benefit future nationwide CHD TTE training and multicentre research.

## Principles of sequential segmental analysis by echocardiography

Whilst it is appreciated that CHD imaging protocols will to some extent vary from centre to centre, sequential segmental analysis within CHD TTE is essential for detailing cardiovascular anatomy and ensuring important pathology is not missed [6]. Concomitant with the advent of TTE, sequential segmental analysis was standardised by Anderson and colleagues and is well established within paediatric imaging practices [8, 16–18]. Principally, this approach removes speculative assumption and permits cardiac morphology to be described into cardiac segments (atriums, ventricles, great arteries) and connections (atrioventricular and ventriculoarterial junctions) in a logical narrative, through identifying salient morphological features. It is recognised that complete segmental and functional assessment is not always feasible by TTE and that there is a need for complimentary multi-modality imaging.

### Situs

**Fig. 1:**
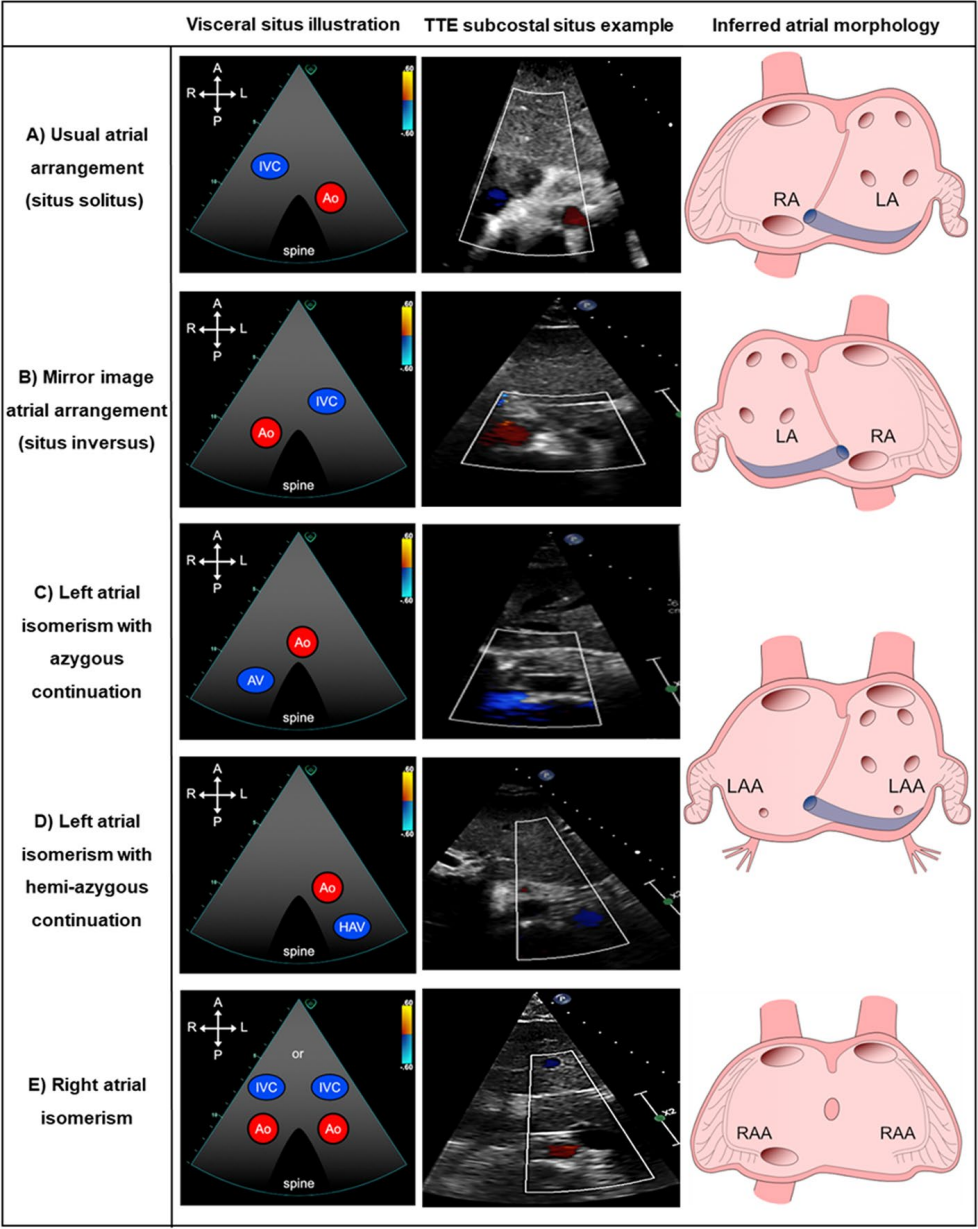
Inferred atrial arrangement from the abdominal visceral situs. **A** Situs solitus: Normal atrial arrangement with right-sided right atrium (RA) and left-sided left atrium (LA). **B** Situs inversus: Mirror-image atrial arrangement with left-sided RA and right-sided LA. **C** Left atrial isomerism (LAI) with azygous continuation. **D** Left atrial isomerism with hemi-azygous continuation. In LAI, venous blood can return to the atria via the right superior vena cava (SVC), direct left SVC insertion or left SVC to coronary sinus (CS). There is direct hepatic drainage to the atriums, the CS is a left-sided structure and is often present, and whilst pulmonary veins often connect normally, they can drain abnormally (i.e. septal malposition or symmetrically to either side of the atrial septum). **E** Right atrial isomerism: cardiac anomalies are often more complex with inherent total anomalous pulmonary venous return, bilateral SVCs, absent CS and typically a common atrium. The concept of two morphologically identical atria/appendages is adopted for diagrammatic educational purposes only. Illustrations modified with permission from Geva L (2021) segmental approach to congenital heart disease. *In Echocardiography in Pediatric and Congenital Heart Disease: From Fetus to Adult,* 3rd edn, ch 3. Eds WW Lai, LL Mertons, MS Cohen, T Geva. Copyright 2022 Wiley-Blackwell

Subcostal imaging should be initially conducted in order to infer atrial arrangement from the abdominal visceral situs (lateralised body arrangement), since direct visualization of atrial morphology by TTE, such as the appendages (right atrium: triangular and broad-based versus left atrium: narrow finger-like) is largely not possible [6]. By TTE, atrial arrangement is principally inferred from the position of the inferior vena cava (compressible and non-pulsatile) and aorta (round, non-compressible and pulsatile) relative to the spine (Fig. 1). If a ipsilateral vein is seen posterior to the aorta, interruption of the inferior vena cava should be suspected and equally if an ipsilateral vein is seen anterior to the aorta, right atrial isomerism should be suspected [19, 20]. It is important to note that veno-atrial drainage and the side in which the atrium is positioned do not define atrial morphology [19]. Alongside atrial arrangement, subcostal imaging can permit gross anatomy (cardiac segments and connections) understanding, if attainable. Although, latter imaging is additive for further detailed assessment, the preferred imaging order of TTE sequential segmental analysis discourages starting from the parasternal window in the CHD patient without an established diagnosis, where the standard adult TTE study commences.

### Cardiac position and cardiac apex

**Fig. 2:**
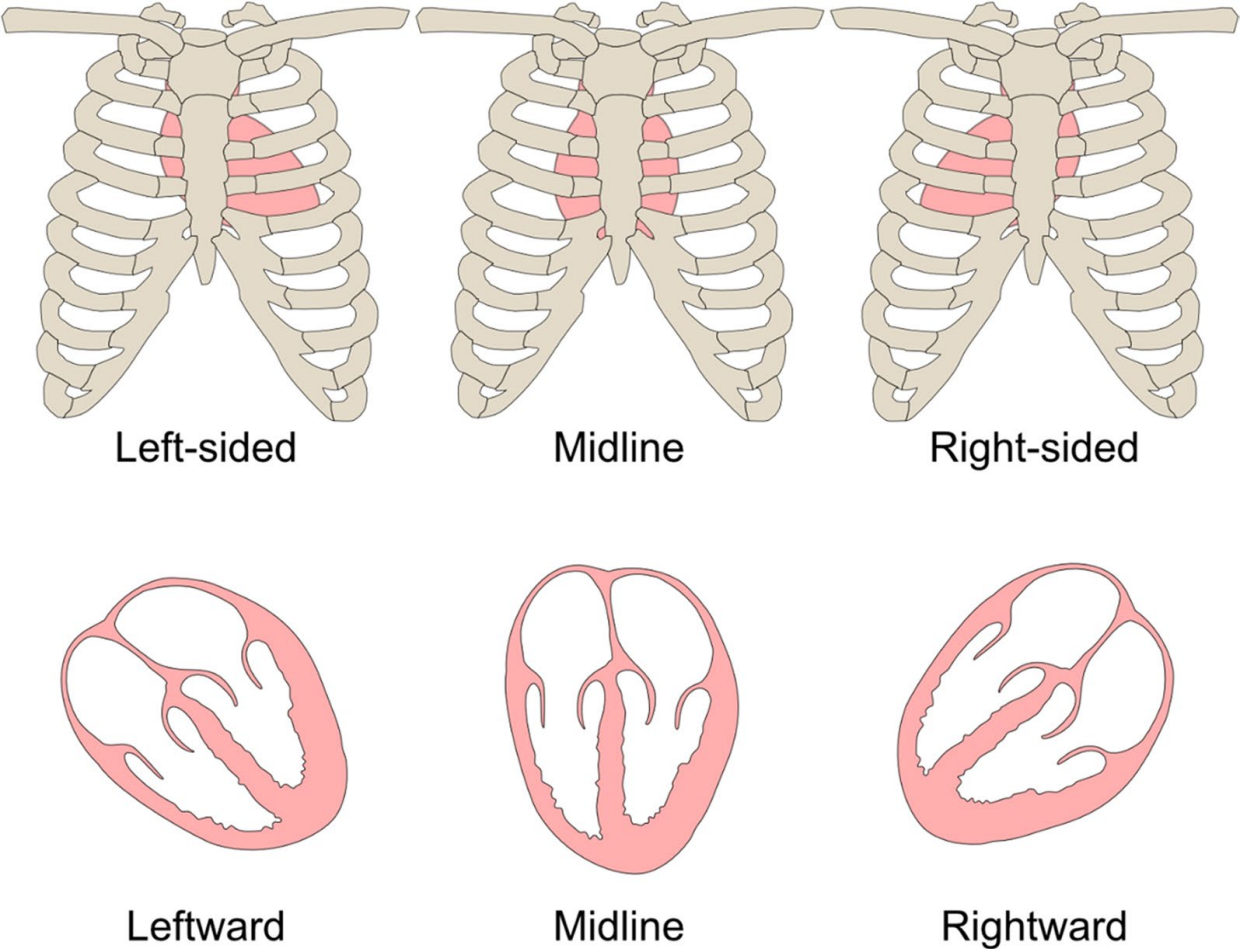
Cardiac position in reference to the thorax and relative position of the apex

Cardiac position should then be defined in reference to which side of the chest the heart lies and the relative position of the apex (Fig. 2). In order to avoid confusing nomenclature such as ‘dextroposition with dextrocardia apex orientation and/or dextroversion’, we advocate a simpler descriptive approach whereby cardiac position is characterised as left-sided, right-sided or midline alongside a leftward, rightward or midline apex. Cardiac position and orientation can be different if the heart is pushed or pulled from an extracardiac abnormality.

### Cardiac chambers and connections

**Fig. 3:**
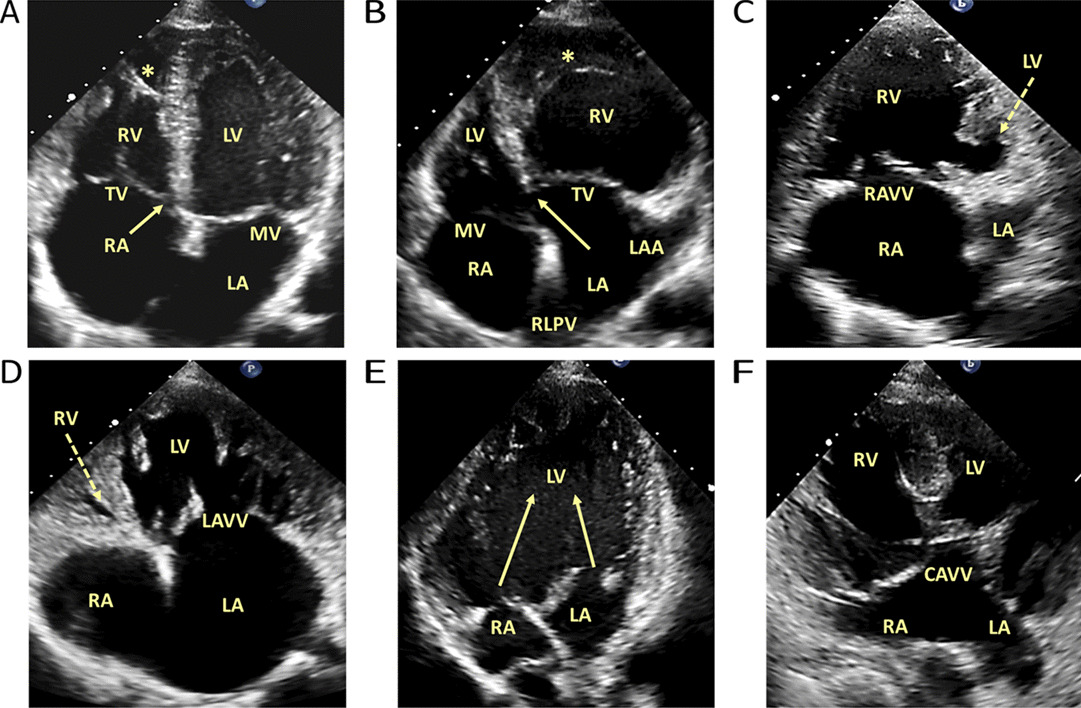
Potential atrioventricular (AV) connections examples. **A** Concordant AV connections. **B** Discordant AV connections. The right ventricle is more trabeculated to the LV and there is a distinct moderator band (*). The arrows in A and B highlight the apical offset of the tricuspid valve, which helps identify the morphological right ventricle. **C** Absent left AV valve/mitral valve. **D** Absent right AV valve/tricuspid valve. The dashed arrows in **C** and **D** identify the hypoplastic/rudimentary ventricle. **E** Double inlet left ventricle connection. The arrows in **E** exhibit the inflow nature of both AV valves to a single left ventricle. **F** Common AV valve (CAVV) connection. All images are examples from usual atrial arrangement (situs solitus) anatomies for convenience. Further variability can exist, which demonstrates the importance of sequential segmental analysis. * = moderator band

The atrioventricular connection and the respective valve morphology should then be examined (Fig. 3). In the normally connected heart, the left and right atriums will connect via individual atrioventricular valves to their respective left and right ventricles, which is described as concordant atrioventricular connections. However, atriums can also connect to an opposing ventricle (discordant atrioventricular connections), not connect at all (absent), or both to the same ventricle (double inlet) [19]. In most biventricular anatomies, unless the atrioventricular junction is guarded by a common valve, the type of atrioventricular valve (i.e. tricuspid or mitral valve) directly aligns with the expected morphological ventricle (i.e. right or left ventricle, respectively). A tricuspid valve is typically more apically inserted with a trileaflet triangular orifice and septal leaflet/chordal insertion into the ventricular septal mass. Whereas a mitral valve typically has a more basal insertion, a bileaflet elliptical orifice with two distinct papillary muscles inserting away from the ventricular septal mass. Confirmation of ventricular morphology can be supported by an awareness that the right ventricle comprises a moderator band, courser trabeculations and three components: inlet, apical and outlet portions (crescentic shape) versus a more cone-like, smooth walled left ventricle morphology. However, caution should be adopted with respect to identifiable TTE features of ventricular morphology, due to the impact abnormal connections or loading conditions can have on their appearance.

**Fig. 4:**
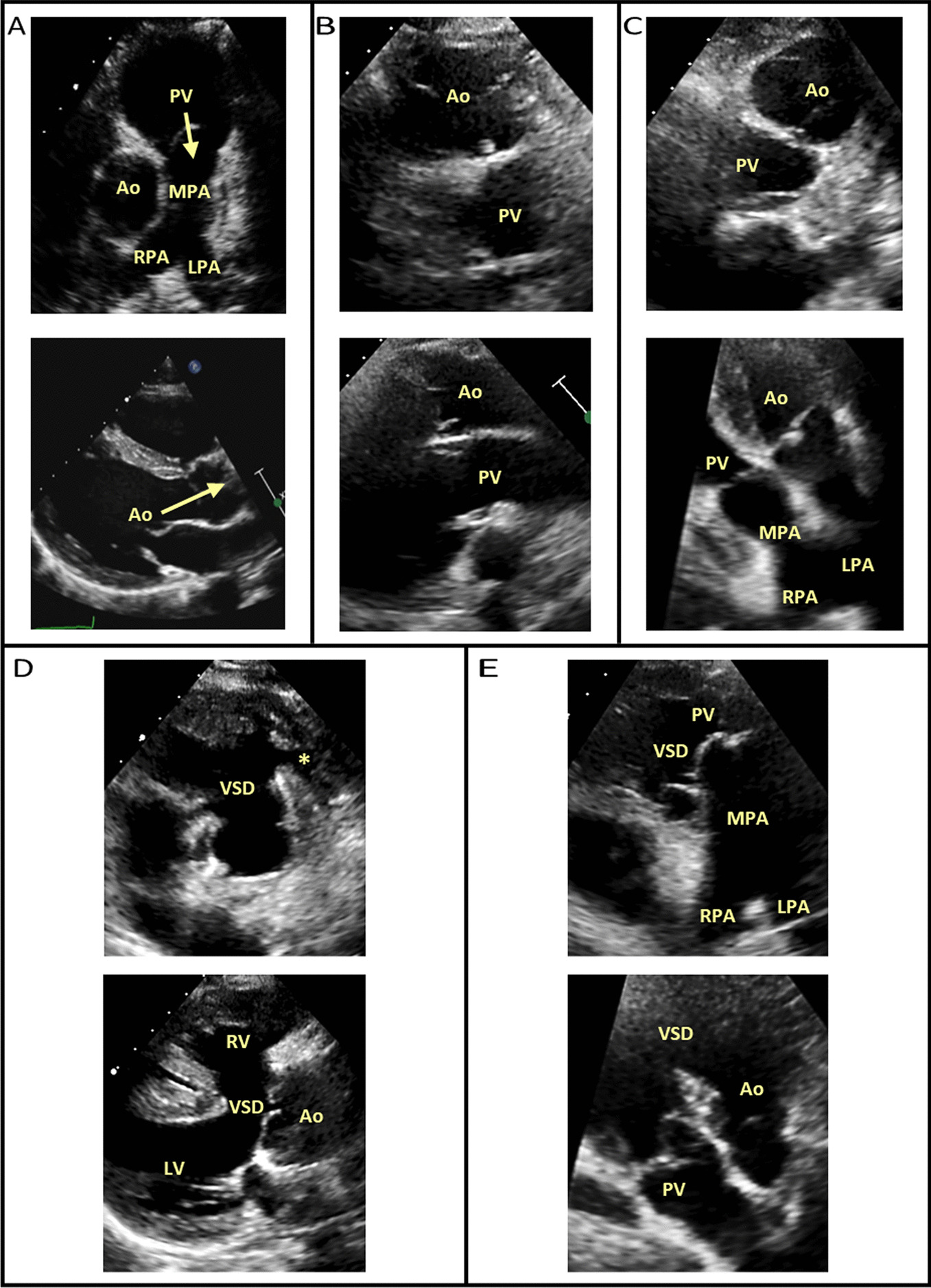
Potential ventriculoarterial (VA) connections examples. **A** Concordant VA connection (pulmonary outflow is anterior and leftward relative to the aorta (outflow roots cannot be viewed simultaneously, arrows identify respective outflow direction). **B** Discordant VA connection— transposition of the great arteries (aorta is anterior and rightward relative to the pulmonary valve in a parallel arrangement). **C** Discordant VA connection— congenitally corrected transposition of the great arteries (aorta is anterior and leftward relative to the pulmonary valve in a parallel arrangement). **D** Absent right VA valve/atretic pulmonary valve connection (*).From the parasternal window, differential diagnosis must be considered when a single overriding outlet is observed, for example pulmonary atresia, tetralogy of Fallot or truncus arteriosus. **E** Double outlet right ventricle. All images are examples from usual atrial arrangement (situs solitus) anatomies for convenience. Further variability can exist, which demonstrates the importance of sequential segmental analysis.

The ventriculoarterial connection and the respective great artery morphology should then be examined (Fig. 4). In the normally connected heart, the left ventricle connects to the aorta, which typically gives rise to the coronary ostia then head and neck vessels. Whilst the right ventricle connects to the pulmonary artery, which typically exhibits bifurcation into respective left and right pulmonary artery branches. These are concordant ventriculoarterial connections with normal spatial orientation, whereby the pulmonary outflow is anterior and leftward relative to the aorta and in short-axis views the aorta is seen enface and pulmonary outflow along its length [6, 19]. However, unless this junction is guarded by a common trunk, great arteries can also connect to opposing ventricles (discordant ventriculoarterial connections), not connect at all (absent/atretic), or arise both completely or mostly from the same ventricle, in which spatial orientation can be highly variable (double outlet) [19].

In the normal heart, the connection between the right ventricle and the pulmonary artery includes a complete ring of muscle (infundibulum). In contrast, the normal left ventricular outflow tract is characterised by partial absence of an infundibulum with fibrous continuity of the mitral valve and the aorta [19]. In the abnormal heart, outlet septum morphology may change. The outlet septum (muscle between the outlets of the heart), which is also referred to as the conal septum, may be deviated into either the subpulmonary or subaortic region to cause obstruction to either outlet of the heart. For example, tetralogy of Fallot is characterised by anterior deviation of muscle (the outlet septum) into the right ventricular outflow tract, causing a variable degree of obstruction. In other types of abnormal ventriculoarterial connection, for example, simple transposition of the great arteries, there is a subaortic infundibulum and fibrous continuity between the mitral and pulmonary valve. In more complex forms of transposition of the great arteries or double outlet right ventricle there may be both subaortic and sub-pulmonary infundibulum with associated loss of continuity between the corresponding atrioventricular valve and great artery. In rare instances of double outlet left ventricle, an outlet septum may exhibit bilaterally absent sub-aortic and sub-pulmonary infundibulum with associated fibrous continuity between the corresponding atrioventricular valve and great artery.

## Image optimisation

It is essential that all TTE studies are optimised. Within CHD, there are particular imaging challenges to consider. An appreciation of these challenges and an awareness of how they can sometimes be overcome will facilitate a comprehensive assessment of the congenital anatomy. Common encounters include variable patient size, chest shape malformations, bespoke positioning of surgical baffles/conduits and/or significant surgical scarring, because of multiple surgeries. These can often be overcome through utilising non-orthodox “off-axis” imaging planes and/or “sweeps” (i.e. using timed captured storage), if deemed necessary, to display and quantify anatomy or pathology. Although the adoption of cardiac “sweeps” within CHD TTE can be advantageous, they should not be over utilised. They are most beneficial with respect to greater appreciation of complex CHD anatomies and relationships.

Detailed guidance on frequency selection, focus, harmonics, gain, compression, colour Doppler, zoom, alongside the adoption of more novel TTE technologies (i.e. 3D and speckle tracking) and general TTE lab set-up (i.e. identifying information, chaperones, etc.) have been addressed in detail in the most recent BSE adult minimum dataset [10]. When low velocity flow is being assessed (e.g. venous flow in Fontan palliated circuits or coronary flow), velocity ranges should reflect this with a low Nyquist limit (i.e. 15–20 cm/s) and decreased wall filters to capture spectral Doppler information. In instances where it is not always possible to attain complete sequential segmental imaging studies in CHD populations (i.e. inadequate imaging “window” and/or un-cooperation), reports should detail this fact, as well as whether this is a follow-up serial surveillance imaging study, which itself can negate the need to perform a “full CHD TTE study” as long as the clinical question remains answered.

## Imaging interpretation, time and reporting

It is strongly recommended to record height, weight (body surface area), heart rate/electrocardiogram, oxygen saturation and blood pressure details in order to maximise sizing, function and haemodynamic quantification. Careful consideration should be given when adopting normative adult reference datasets [15], as these are not directly applicable to the entire spectrum of the CHD cohort. Typically, more emphasis should be placed on the patient’s baseline and/or serial comparative TTE studies, which often act as their own normative/acceptable range data. Furthermore, whilst Z-scoring is routine in paediatric TTE practices in order to appreciate cardiovascular allometric scaling, it is accepted that it is not routine within ACHD TTE environments.

Sufficient time should be allocated to each CHD TTE study. Additional time may be required to study a complex anatomy, pathology, haemodynamic or functional state. Consideration should also be given to the potential amenability of a patient. Conversely, an uncomplicated CHD lesion or follow-up surveillance CHD TTE exam may require less time. Additional post-processing time for advanced imaging techniques, such as 3D and speckle tracking, that have been shown to benefit assessment of cardiac morphology, physiology, pathophysiology, and function, should be permitted [7, 21–23]. Incorporation of advanced imaging techniques into departmental practices is of growing importance in order to develop echocardiography expertise and to aid future research and normative dataset development.

The reporting of a patient’s first CHD TTE and/or pre-admission TTE study, when a “full CHD TTE study” is typically performed, should mirror that of the adopted sequential segmental imaging approach (Table 4) [6, 7]. A follow-up report is typically more focused, reflecting the nature of CHD TTE surveillance imaging; this being based on the patient’s specific anatomical and functional complexities. Within ACHD settings, TTE reports are typically pre-formatted to reflect standard adult cardiology practice [10]. Whilst this reporting style is adequate for most biventricular anatomies, if all cardiac segments and connections remain described, it is not advisable for complex/unrepaired congenital pathologies, systemic right ventricles, or Fontan palliated populations.

## Specific considerations when interpreting the congenital echocardiogram

It is important to recognise the situations where normative values and practices of standard adult TTE are not applicable in the CHD patient. When the size, morphology, haemodynamic loading condition and/or position of the cardiac ventricles are abnormal, assumptive interpretations of standard adult TTE are invalid [7]. Common examples encountered include:

### The systemic right ventricle i.e. congenitally corrected transposition of the great arteries and post Mustard’s or Senning’s atrial-switch repair

Besides serial comparison, TTE assessment remains largely qualitative. Tricuspid regurgitation cannot be implemented in pulmonary pressure probability (i.e. use sub-pulmonic mitral regurgitation, if present) and the use of geometric Simpson’s ejection fraction method on the systemic right ventricle is invalid [24]. Careful assessment for tricuspid valve regurgitation is needed in the systemic right ventricle as this carries prognostic and clinical significance. The usual indicators of severity remain carefully adopted as detailed in the BSE valvular guidelines as they are not directly applicable [11]. The primary mechanisms that make systemic tricuspid valve regurgitation a common feature include irregular interventricular septal confirmation, leaflet tethering from right ventricular dilation and/or dysplastic leaflet displacement resembling ‘Ebstein-like’ malformation that is often exhibited in congenitally corrected transposition of the great arteries. With respect to diastology in atrial-switch repair anatomies, baffle compliance (i.e. often stiff and non-contractile) will abnormally impact inflow characteristics. Thus, although important, standard inflow diastolic parameters generally remain non-applicable [25, 26].

### Unrepaired univentricular physiology and Fontan palliations

These offer further complexities with respect to geometry and contractility compared to the structurally normal heart and although TTE assessment remains the mainstay, TTE functional assessment is essentially qualitative with standard measurement parameters only serving to aid serial comparisons, rather than permitting graded quantification. Consequently, there remains an important role for the visual assessment (‘eyeballing’) of the single /uni- ventricle function. Diastolic assessment is also erroneous in Fontan patients due to being pre-load deprived [27]. There is additionally a lack of any reliable normative data given the morphological heterogeneity of this group.

### Cardiac surgery

Where cardiac surgery has taken place, abnormally reduced myocardial motion may be misleading to overall function. For example, basal septal parameters in those with ventricular septal defect patch repair will be reduced, but overall function may remain normal as there isn’t necessarily a primary issue with the myocardium.

### Atrioventricular anomalies i.e. atrioventricular septal defects

Valvular quantitative methods are often invalid. For example, linear areas of regurgitation do not apply in cleft anatomy, whilst residual shunting (including left ventricle to right atrium), which are recognised sequalae, alter the haemodynamic conditions for valid quantification.

### Shunts

Many CHD patients possess intracardiac shunts, which must be considered with respect to functional and haemodynamic TTE quantification. For example, underestimation of valve obstruction will occur if a chamber is enabled to “blow-off” through a shunt, and hence chamber pressure will not increase to generate high gradients (i.e. ventricular septal defects and aortic stenosis or atrial septal defects and mitral stenosis). Similarly, it should be understood that in the presence of an isolated shunt defect, inflow gradients may be falsely elevated in the context of increased veno-atrial flow (i.e. ventricular septal defect and mitral inflow).

## Image orientation

There is a wide spectrum of adopted practices in relation to the orientation of the CHD TTE study. A CHD TTE study may be performed displaying superior and anterior structures at the top of the imaging screen, the apex at the bottom and rightward structures on the left side of the imaging screen. This demonstrates cardiac structures in their ‘anatomical’ orientation (does not apply to the parasternal long axis). Alternatively, CHD TTE may reflect working practices purely from an ‘adult’ orientation, with the apex positioned at the top of the imaging screen. It should be noted that “adult” orientated TTE is now a relative outlier compared to other cross-sectional imaging modalities, with the reason for its adoption being somewhat historic due to the inability of early ultrasound technologies to modify the image screen. Irrespective of the study orientation, the CHD TTE study should be presented in a way that allows the operator and reviewing clinicians to correctly interpret them. This may reflect a combination of both orientations, particularly when trying to better understand complex congenital anatomies, in which it is recognised that ‘anatomical’ orientated TTE imaging can aid sequential segmental cardiac morphology interpretation. This is especially apparent with the initial subcostal window. It is recognised that many subcostal CHD TTE views are not routinely performed in the adult TTE study, thus this will present new learning for those in CHD TTE training, when attainable. We therefore recommend, particularly within ACHD services, that CHD TTE should seek to offer training in the acquisition and interpretation of both orientations. The TTE imaging within the CHD TTE BSE guidelines will reflect ‘anatomical’ orientation for the subcostal views and ‘adult’ orientation for the apical views.

## The practical sequential segmental transthoracic imaging congenital heart disease guideline

Table 1 outlines a proposed sequential segmental TTE practical guideline in the CHD patient. It encompasses the recommended dataset to be undertaken (**in bold**) and is structured in the preferred order for a complete anatomical and functional sequential segmental CHD TTE study. It outlines the specific view, modality of assessment, anatomy to assess and measurements to be taken alongside example TTE imaging. Abbreviations used within Table 1 are outlined in Table 2. The CHD TTE imaging guideline is summarised in Table 3 (one-page). An example sequential segmental TTE report is also provided (Table 4). This guideline will not duplicate functional guidance already detailed within BSE adult TTE guidelines but will detail the areas where these guidelines may have limitations or are not directly applicable in the CHD setting [10–15].

**Table 1:** The practical guideline for performing a comprehensive sequential segmental TTE in the CHD patient

**View** (modality)	**Anatomy**	**Measurement ± explanatory note**	**Image**
**Subcostal: **Transverse / axial plane (2D & CFD)	**Visceral / inferred atrial situs**	Indicator @ ~ 3 o’clock **Situs view with IVC, Ab. Ao. and spine in cross section to assess their relative position** **Normal (situs solitus):** Aorta to left of spine, IVC rightward (± slightly anterior). Rotation and angulation to exhibit long axis of the IVC can be utilised to demonstrate IVC draining into RA	
**Subcostal: **Longitudinal / sagittal plane (2D & CFD)	**IVC in long axis** Hepatic vein	Indicator @ ~ 12 o’clock. In situs solitus, slightly rightward tilt for IVC **IVC:** – *As detailed by BSE minimum dataset *[10] – **Presence, connection, size, RAP estimation **[15] – MM imaging can also be adopted, if perpendicular **Normal:** Hepatics return to the IVC, near IVC/RA junction. CFD and PWD in the dominant hepatic vein can be used to inspect for systolic dominance or diastolic reversal (which can infer TR severity and/or restrictive RV physiology)	
	**Ab. aorta in long axis**	**Visually assess for pulsatility and for any abnormal flow pattern.** From the IVC view, keep indicator at same position and sweep to the left of the patient (in situs solitus) to open Ab. Ao Note: Depending on probe position celiac trunk/superior mesenteric artery may also be visualised	
**Subcostal: **Longitudinal / sagittal plane (PWD)	**Ab. aorta**	**Assess for pulsatility to confirm Ab. Ao. and assess for any abnormal flow pattern.** In situs solitus, leftward tilt for Ab. Ao **Normal:** Triphasic signal. Some patients may exhibit reduced aortic elastic compliance and loose triphasic profile, but still be considered normal (i.e. older populations, aortic arch repair) **Differential diagnosis:** If continuous pulsatile forward flow, consider; – Contamination with celiac trunk/superior mesenteric artery flow – If also blunted/spectral broadening, consider upstream obstruction, i.e. coarctation If holo-diastolic diastolic flow reversal, likely significant aortic regurgitation, but also consider (particularly if within paediatric practices); – PDA with significant pulmonary “run-off” – Aortopulmonary window defect – Major aortopulmonary collateral arteries – Unrepaired truncus – Large Blalock-Taussig shunt – Duct dependent flow; i.e. AoV atresia, interrupted aortic arch	
**Subcostal: **Long axis / coronal plane (2D & CFD)	**Heart orientation & position**	Indicator @ ~ 3 o’clock **Establish laterality/cardiac position and apex position of the heart** **Normal:** Left-sided with leftward apex Entire cardiac morphology, particularly with optimal windows may be appreciated; – **Veno-atrial drainage/connection**—Detailed throughout, but if anomalous veno-atrial return (i.e. shunt), – **Atrium morphology**—Can be appreciated from multiple windows. Detailed in PSAX window – **Atrioventricular connection and function**—Can be appreciated from multiple windows. Detail in A4C description (Fig. 3) – **Ventricular morphology and function**—Can be appreciated from multiple windows. Detailed in A4C description. * = septal leaflet chordal attachment of the tricuspid valve – **Shunts**—ASD, VSD, PDA – **Ventriculoarterial connection and function** – Can be appreciated from multiple windows (subcostal, PSAX, apical); **Normal:** Keeping indicator at @ ~ 3 o’clock, tilt anterior to demonstrate outflows sequentially. A sweep stored loop is valuable. Pulmonary outflow is anterior and leftward of the aortic outflow (** →**). Branching pattern may help decipher outflow morphology. Other spatial arrangements exist (Fig. 4)	
	**Pericardium**	Pericardial effusion assessment	
	**Atrial septum**	Indicator @ ~ 4/5 o’clock ± anterior tilt **Lengthen IAS. Assess for ASD** Can also visualise RLPV/RUPV and LLPV Image may require optimising to ensure adequate resolution (frame rate), particularly in adults, in whom the heart is often imaged in the far-field	
**Subcostal: Bicaval **Short axis (2D & CFD)	**Atrial septum** **RSVC** **RAA**	Indicator @ 5/6 o’clock **Exhibit veno-atrial drainage/connection** **Assess IAS. Assess for ASD** **Note:** Generally, the recomended image plane (or slightly modified) to interrogate for inferior and superior sinus venous shunt defects. A high right parasternal view with the probe in a super-inferior orientation can also be adopted. Eustachian valve (IVC) is often seen and if prominent directs flow towards the IAS Abnormal diastolic flow reversal may be seen with restrictive RV physiology and/or with significant TR severity May be appreciated on lateral aspect of RA, towards RSVC ostium. Detailed in PSAX window description	
	Ventricular morphology Full heart sweep **Pulmonary outflow**	Look for chordal septal attachment of TV (*) to help infer RV morphology and equally MV anatomy & apparatus (this can be confirmed in multiple windows, particularly when there is a sub-optimal subcostal window, i.e. most adult cohorts) **Pulmonic outflow assessment;** –*Inspect and interrogate for obstruction* (*sub-, valve, supra-*)* and/or regurgitation as* *detailed by BSE minimum dataset *[10, 13, 15] **Note**: The pulmonary outflow is sometimes best aligned for interrogation from the subcostal window. This is particularly true for the typically anterior located RV-PA conduits, that are often not always easily identified Can assess full cardiac morphology, as previously detailed. Sweep laterally from bicaval to LV apex (akin to PSAX window)	
	MV/LAVV	Detailed in PSAX window. Qualitative assessment of leaflet morphology, thickness, excursion, regurgitation, chordae and papillary morphology/apparatus abnormalities **Normal MV:** The AMVL is parallel to the ventricular septum as depicted (versus perpendicular orientation to the ventricular septum for LAVV anatomy)	
**Subcostal:** RV inflow/outflow (2D & CFD) RAO view AKA “ToF View”	**AoV, IAS, RA, TV, RV, RVOT, PA, MPA & PA branches**	Indicator @ 1 o’clock. A similar RV inflow/outflow window (** →**) can be replicated from a modified apical RV 3 chamber window Offset between the AoV and PV can often be well appreciated, with PV morphology being superior **Assess for VSD, abnormality of outlet septum, RVOT obstruction and/or double chambered RV anatomy; ** **Normal (sub-pulmonary):** The right ventricle—pulmonary artery junction has a complete circumferential muscular outlet-septum boundary, resulting in discontinuity between the tricuspid valve and pulmonary outflow. The left ventricle—aortic outflow does not, which results in aortomitral continuity	
**Apical**: A4C (2D & CFD)	Full heart sweep	State sweep direction: Can assess full cardiac morphology, as previously detailed **Note:** Although IAS mobility and competence can be appreciated, caution must be adhered to with regards to potential ASD interrogation, sizing and shunt direction due to lateral resolution “drop out” artefact. Assess for ASD; A modified A4C where the septum is orientated more perpendicular will help minimise error. * = moderator band ** → ** = atrioventricular valve offset	
	**Pulmonary veins:** RLPV/RUPV & LLPV	**Pulmonary vein identification;** –*As detailed by BSE minimum dataset *[10, 15] RLPV is most likely seen when adjacent to the IAS in A4C with RUPV more likely to be visualised when more anteriorly tilted LLPV is generally well appreciated from A4C **Note:** Identification and interrogation for pulmonary veins is important with respect to; –Anomalous drainage/connection –Isolated obstruction –Atrioventricular valve regurgitation –Diastolic function	

A4C (PWD/CWD)	Pulmonary veins	Pulmonary vein interrogation; –*A higher sweep speed (i.e.* > *75 mm/s) will facilitate greater accuracy of atrial reversal duration measurement. As detailed by BSE minimum dataset *[10, 15]	
	**Atrio-ventricular connection & ventricular morphology**	**The atrioventricular valve directly correlates to the morphological ventricle;** – **Left ventricle = MV: Inserts further from apex** – **Right ventricle = TV: Inserts closer to the apex (A)** – **Atrioventricular valve offset:** If offset is reversed (B), the TV is abnormally apically inserted (Ebsteins: > 8 mm/m2, C), or not offset (D), question underlying atrioventricular anatomy (Fig. 3)	
		**Morphology can then further be interrogated from the following;** – Leaflet morphology (in absence of common atrioventricular valve): TV: Trileaflet triangular orifice with septal leaflet attachment into the ventricular myocardium MV: Bileaflet elliptical orifice with typically two distinct papillary muscles inserted antero-laterally and postero-medially (SAX and A2C windows) – Moderator band: Increases likelihood of RV morphology – Wall smoothness: RV more likely to have a courser septal surface with apical trabeculation versus a smooth walled LV – Ventricular shape: RV is crescentic versus cone-like LV appearance	
A4C (2D & CFD)	**MV/LAVV**	**Caution must be adhered to when assessing systemic TVs or LAVVs with respect to BSE adult quantification data, as it will not be directly applicable** **Systemic regurgitation assessment;** – *If MV, as detailed by BSE guidelines *[11, 15] –Dominant MV scallops in A4C: A3/A2 & P1	
	**Left atrium**	**Systemic stenosis assessment;** – *As detailed by BSE guidelines *[11, 15] **LA size (end systolic volume): Biplane** – *As detailed by BSE minimum dataset *[10, 15]	
	**MV/LAVV**	**Caution must be adhered to when quantifying regurgitation and/or stenosis in the presence of intracardiac shunts** **Systemic regurgitation assessment;** –*As detailed by BSE guidelines *[11, 15]	
		**Systemic stenosis asessment;** –*As detailed by BSE guidelines *[11, 15]	
A4C (PWD)	**MV/LAVV**	**Caution must be adhered to when quantifying inflow with respect to atrial switch biventricular repair anatomies or univentricular physiology** **Systemic inflow;** –*As detailed by BSE minimum dataset *[10, 11, 15]	
A4C (TDI/MM)	**Basal lateral and basal septal annulus**	**Caution must be adhered to when quantifying TDI with respect to atrial switch biventricular repair anatomies or univentricular physiology** **Systemic systolic and diastolic assessment;** -*As detailed by BSE minimum dataset *[10, 11, 15] **Note:** Limited utility when RWMA are present or expected (i.e. VSD patch, atrioventricular valve repair/replacement)	
	**Lateral annulus**	**MAPSE** –*As detailed by BSE minimum dataset *[10, 15]	
A5C (2D & CFD)	**Outflow/root**	**Ventriculoarterial connection and function; ** Tilt anterior to demonstrate outflows sequentially. A sweep stored loop may be of value **Normal:** Pulmonary valve/outflow is anterior and leftward of the aortic valve/outflow. Branching pattern may help decipher outflow morphology. Spatial orientation of the ventriculoarterial connections can be appreciated (Fig. 4) **Systemic outflow assessment;** – Inspect and interrogate for obstruction *(sub-, valve, supra-) and/or regurgitation as* detailed by BSE minimum dataset [10, 12] **Normal:** Aortomitral continuity as sub-pulmonary outlet septum morphology In atrioventricular septal defect anatomies, there is unwedging of the aorta with absence of atrioventricular—ventriculoarterial continuity. Aortomitral discontinuity will also be noted with respect to systemic RV physiology	
	Coronaries	In addition to PSAX window, coronary ostia and their course can sometimes be appreciated when interrogating the aortic outflow with further anterior modified angulations ( **→**)	
A5C (PWD/CWD)	**Outflow/root**	**Systemic outflow assessment;** -*Inspect and interrogate for obstruction* (*sub-, valve, supra-*)* and/or regurgitation as* *detailed by BSE minimum dataset *[10, 12, 15] Note: Supra-valvular outflow obstructions are often well visualised and quantified from suprasternal and right parasternal windows	
A4C 2D Zoom	**Left ventricle**	**Systemic LV assessment;** –*As detailed by BSE minimum dataset *[10, 15] –A4C LV (** →**): IS: Infero-septum wall AL: Antero-lateral wall Measures should be indexed to BSA or Z-scored (if paediatrics)	
A2C (2D, CFD, PWD, CWD), 2D Zoom	**Left ventricle** **Left atrium**	**Systemic LV assessment;** –*As detailed by BSE minimum dataset *[10, 15] –A2C LV (** →**): I: Inferior wall A: Anterior wall **LA size (end systolic volume); Biplane** – *As detailed by BSE minimum dataset *[10, 15]	
	**MV/LAVV**	If MV: P3/A2 (enface)/P1. Assess for stenosis* and/or regurgitation as* *detailed by BSE guidelines *[10, 11, 15]*.* **Assessment of papillary muscle number and location with modified angulations** Note: X-plane enface imaging or 3D helpful when interrogating anatomy [11], especially if LAVV	
	**Pulmonary veins**	LUPV is seen adjacent to LAA, separated by the coumadin ridge.	
	Coronary sinus	RLPV/RUPV may also be appreciated with modified angulation CS may also be appreciated enface, adjacent to the basal inferior wall, within the atrioventricular groove	
	Ab. aorta	Further posterior angulation will demonstrate the Ab. Ao. in its long axis	
A3C (2D, CFD, PWD, CWD), 2D Zoom	**Left ventricle**	**Systemic LV assessment;** – *As detailed by BSE minimum dataset *[10] – A3C LV (** →**): IL: Infero-lateral wall AS: Antero-septum wall	
	**MV/LAVV** **Outflow/root**	IF MV: Likely A2/P2. Assess for stenosis* and/or regurgitation as* *detailed by BSE guidelines *[10, 11, 15] **Systemic outflow assessment;** –*Inspect and interrogate for obstruction* (sub-, valve, supra-)* and/or regurgitation as* *detailed by BSE minimum dataset *[10, 12, 15]	
A4C RV (2D & CFD, MM, TDI)	**Right ventricle** **RA**	**Subpulmonic RV assessment;** - *As detailed by BSE minimum dataset *[10, 14, 15] - If RV: Lateral RV “free” wall Note: Systemic RV assessment is largely qualitative, with measurement parameters adopted for longitudinal follow-up comparison **RA size (end systolic area);** -*As detailed by BSE minimum dataset *[10, 15]	
		**Obtain a RV focussed view to allow entire RV free wall to be interrogated and RV dimensions to be measured from.** *As detailed by BSE minimum dataset *[10, 14, 15]	
	**TV/RAVV**	**Caution must be adhered to when assessing subpulmonic MVs or RAVVs with respect to BSE adult TV quantification data, as it will not be directly applicable** **Subpulmonic TV inflow stenosis/regurgitation;** – *Assess as detailed by BSE guidelines *[13, 15] – If TV: Likely anterior (A) & septal (S) leaflets – Posterior (P) leaflet if CS angulated in view Note: X-plane enface imaging or 3D helpful when interrogating anatomy [13]	
	Coronary sinus	With posterior angulation, CS can be appreciated, draining back into the RA within the left posterior atrioventricular groove. *In LSVC anatomy, a dilated CS is often seen (right example). Note: The LSVC is a left atrium morphological structure* **If CS is unroofed/fenestrated, thus shunting ** **physiologically, haemodynamically will act as an ASD with left to right shunting**	
	RSVC	Sometimes noted very anterior	
	RAA	Broad based orifice and anterior. Christa terminalis (prominent muscle bar separating RSVC—RAA) may be seen	
	Eustachian valve	** →** = Eustachian valve (EV)/ridge can sometimes be seen and if may be noted to change inflow profile. It is typically a relatively rigid structure, inserting more caudally (infero-posterior), but can also be mobile and fenestrated. It is different to the Chiari network due to its insertion point, which inserts superior-anterior This is a normal anatomical variant. Possible erroneous pathologic interpretation typically includes mass lesion or Cor triatriatum dexter	
Modified apical RV 3 chamber Inflow/outflow (2D & CFD)	RA, TV, RV, RVOT, PA, MPA & PA branches	Anterior probe tilt with indicator rotated between 12–2 o’clock, akin to the subcostal RV inflow-outflow window (** →**) Depending on rotation of probe, aortic valve may be seen enface [13], if TV: – Adjacent to AoV and no septum—anterior (A) TV leaflet – Liver noted/inferior RV wall—posterior (P) TV leaflet Offset between the AoV and PV can then be appreciated with PV morphology being superior **Assess for abnormality of tricuspid valve (i.e. Ebstein’s), VSD, outlet septum, RVOT obstruction and/or double chambered RV anatomy**	
A4C RV (PWD/CWD)	**TV/RAVV**	**Caution must be adhered to when quantifying regurgitation and/or stenosis in the presence of intracardiac shunts** Est. RVSP = TR Vmax + RAP – If no valvular stenosis, regurgitation, or downstream stenosis (i.e. branch pulmonary arteries), use as surrogate for PASP Note: RVSP/PASP will be underestimated with severe/free TR and should not be relied upon	
		**Subpulmonic TV stenosis/regurgitation;** – *Assess as detailed by BSE guidelines *[10, 13, 15]	
Modified A4C PV (2D, CFD, PWD, CWD),	Outflow/root	Ventriculoarterial connection and function; Tilt further anterior than A5C to demonstrate pulmonary outflow, if required. In some patients, branch pulmonary arteries can also be appreciated Typically, a rib-space higher with lateral or medial modification will optimise this pulmonary outflow window, which can be fully examined **Normal:** Pulmonary valve/outflow is anterior and leftward of the aortic valve/outflow. Branching pattern may help decipher outflow morphology. Spatial orientation of the ventriculoarterial connections can be appreciated (Fig. 4)	
Left PLAX (2D & CFD)	Full heart sweep	State sweep direction: “VSD sweep” Can assess full cardiac morphology, as detailed in previous sweep descriptions **Note: Parasternal imaging often exhibits best VSD visualisation and quantification** **Ventriculoarterial connection; ** Pulmonary valve/outflow is anterior and leftward of the aortic valve/outflow. Branching pattern may help decipher outflow morphology. Spatial orientation of the ventriculoarterial connections can be appreciated (Fig. 4)	
Left PLAX (2D & CFD)	**Pericardial/pleural space** **MV/LAVV** **LA**	Increase scan depth **Assess annulus, mobility, thickness, calcification, supra or sub valvular apparatus** **Systemic stenosis/regurgitation assessment;** – *If MV, measures as detailed by BSE guidelines *[10, 11, 15] MV scallops usually demonstrated are; * Standard PLAX: A2 & P2* * Modified RV inflow tilt: A3 & P3* * Modified RV outflow tilt: A1 & P1* * MM may help demonstrate pathology* Note: X-plane enface imaging or 3D helpful when interrogating anatomy [11], especially if LAVV	
	**Left ventricle**	**Systemic LV assessment;** – *As detailed by BSE minimum dataset *[10, 15] – PLAX LV (** →**): IL: Infero-lateral wall AS: Antero-septum wall	
	Coronary sinus	CS (enface): Posterior within left posterior atrioventricular groove. If dilated, a high index of suspicion is warranted for persistent LSVC anatomy	
	**Prox. RVOT**	Note: Ab. Ao. is seen posterior, outside of the pericardium *As detailed by BSE minimum dataset *[10, 15] If RV: Anterior RV wall	
	Pulmonary veins	LLPV: Adjacent to inferolateral LV annulus Other veins may also be interrogated with modified imaging windows	
Left PLAX: 2D Zoom (2D & CFD)	**LVOT/outflow**	**Systemic outflow assessment;** – *Inspect and interrogate for obstruction* (*sub-, valve, supra-*)* and/or regurgitation as* *detailed by BSE minimum dataset *[10, 12, 15] **Normal:** If trileaflet aortic valve: right coronary cusp (RCC) is seen anteriorly, extending from ventricular septal aspect. Depending on tilt, either non (NCC)- or left- coronary cusp may been seen posteriorly with continuity to the AMVL, due to normal outlet (sub-pulmonary) septum morphology * = sub-aortic ridge	
	**Root**	**Aortic root appearance and dimensions. Moving up a rib-space/superiorly will often optimise view** **Outflow measurements should be made as detailed by BSE minimum dataset***,* **documenting method of quantification (i.e. edge: inner to inner [i2i] or leading to leading [L2L] and timing: end diastole or end systole) alongside absolute values;** – **BSE Height index (mm/m)—end diastole **[15] – Z-score (label which dataset) – BSA correction (cm/m2): i.e. Turners	
	RCA	RCA ostium may be appreciated arising from the sinus. The RCA typically courses rightward (in PLAX) behind the pulmonary artery and below the right atrial appendage along the right atrioventricular groove Note: High take-off (superior to sinus) may also be appreciated as a normal variant	
Left PLAX: RV inflow (2D & CFD)	**RA** **TV/RAVV** Atrial septum	**Inflow stenosis/regurgitation;** – *As detailed by BSE minimum dataset *[10, 15] – **Note: A PMVSD jet may contaminate TR** The beam tilt will dictate which TV leaflets are likely visualised [10, 13, 15], if TV; – Anterior (A) and posterior (P) leaflets when LV/ventricular septum and CS are no longer visualised – Anterior and septal (S) leaflets when ventricular septum and CS ostia to RA noted Note: X-plane enface imaging or 3D helpful when interrogating anatomy, especially if RAVV A modified “RV inflow” with more lateral and caudal probe positioning (i.e. a rib space lower) can exhibit IAS. Assess for ASD	
	**Right ventricle** **IVC, CS**	**RV size and function **[10, 13] When LV/ventricular septum is no longer visualised, RV inferior wall is seen adjacent to diaphragm/liver with contralateral RV anterior wall noted If RV: A: Anterior RV wall I: Inferior RV wall Can be identified with modified inflow tilt. Remnant Thebesian valve from the CS (*), Eustachian valve from the IVC (#) and/or Chiari network (mobile net-like structure) may be identified and noted to change the inflow profile. It is typically a relatively rigid structure, inserting more caudally (infero-posterior), but can also be mobile and fenestrated. It is a normal anatomical variant. Possible erroneous pathologic interpretation typically includes mass lesion or Cor triatriatum dexter	
	**TV/RAVV**	**Inflow stenosis/regurgitation;** – *As detailed by BSE minimum dataset *[10, 13, 15] – **Note: a PMVSD jet may contaminate TR**	
Left PLAX: RV outflow (2D & CFD)	**Distal RVOT** **PV, MPA & PAs**	Tilt superior and centralise **Pulmonic outflow assessment;** – *Inspect and interrogate for obstruction* (*sub-, valve, supra-*)* and/or regurgitation as* *detailed by BSE minimum dataset *[10, 13, 15] **Ventriculoarterial connection;** Pulmonary valve/outflow is anterior and leftward of the aortic valve/outflow. Branching pattern may help decipher outflow morphology. Spatial orientation of the ventriculoarterial connections can be appreciated (Fig. 4) Notes: –LPA is left-sided in PLAX –PDA can sometimes be examined here if Ductal view is non-obtainable	
Left PLAX: RV outflow (PWD/CWD)	**Distal RVOT** **PV outflow, MPA & PAs**	**Pulmonic outflow assessment;** – *Inspect and interrogate for obstruction* (*sub-, valve, supra-*)* and/or regurgitation as* *detailed by BSE minimum dataset *[10, 13, 15]	
	Can also do in PSAX	**Measures;** –**Sub-valvular PWD (Vmax, VTI, SV).** Use PWD “step-through” if suspected sub- or supra-valvular obstruction – **Sub-pulmonic obstruction CWD (Vmax, VTI, mean PG)**	
		– **Sub-pulmonic regurgitation CWD (density, contour, PHT, PR Vmax, PR Vend, PR index)** – Est. Mean PAP: PR Vmax + RAP – Est. End diastolic PAP: PR Vend + RAP – If notching is noted: likely raised pulmonary pressures Note: Severe PR is likely if flow reversal is noted on CFD and Doppler within the branch PAs	
Left PSAX: (2D & CFD)	Full heart sweep **Pericardial / pleural space**	State sweep direction: “VSD sweep” Can assess full cardiac morphology, as detailed in previous sweep descriptions In order to maintain adequate frame rate, it is recommended CFD “box-size” is reduced to cover myocardial lateral and septal aspects individually when inspecting for VSDs **Note: Parasternal imaging often exhibits best VSD visualisation and quantification** Increase scan depth	
Left PSAX: AoV level (2D & CFD)	**Aortic valve** RVOT, PA, MPA & PA	**Qualitative assessment of cusp morphology and function** – *As detailed by BSE minimum dataset *[10] **Spatial orientation of the ventriculoarterial connections can be appreciated (Fig. **4**)** **Normal:** Pulmonary valve/outflow is anterior and leftward of the aortic valve/outflow. Branching pattern may help decipher outflow morphology. RV inflow-outflow demonstrated (** →**)	
	**Coronary origins**	Coronaries in almost all cases arise from the aortic sinuses facing the pulmonary valve. The non-facing sinus/non-coronary cusp is always the cusp adjacent to the IAS **Use coronary specific imaging settings (i.e. high frequency, low compress, increased 2D gain, zoom). If normal, they arise slightly superior (i.e. up a rib-space) to the respective cusps. By TTE;** – **LCA:** indicator ~ 2/3 o’clock (sometimes further clockwise rotation is warranted) with ostium originating typically at 3 o’clock and courses rightward (in PSAX) before bifurcating into left anterior descending (upward in PSAX: #) and left circumflex arteries (downward in PSAX: *) – **RCA:** indicator ~ 1 o’clock with ostium originating typically at 11 o’clock and coursing leftward (in PSAX)	
		For increased diagnostic accuracy (i.e. to rule out erroneous pericardial fold/coronary vein), origins can be demonstrated with CFD (reduced Nyquist scale), exhibiting predominantly diastolic antegrade flow in the structurally normal heart without known occlusive coronary artery disease	
Modified left PSAX: AoV level (2D & CFD)	Atrial septum **RA** **TV/RAVV**	**A focused image is recommended. Assess for ASD** **Inflow stenosis/regurgitation;** – *As detailed by BSE minimum dataset *[10, 13, 15] – **Note: A PMVSD jet may contaminate TR** If TV: Leaflet cusps will vary depending on level of valve interrogation [13] Note: X-plane enface imaging or 3D helpful when interrogating anatomy [13], especially if RAVV	
	**Atrium morphology** LAA	**Atrial arrangement is largely inferred from the abdominal visceral situs as direct visualisation of both atrial appendages (particularly RAA) is challenging by TTE. Therefore, site of the appendages is mostly complimentary** (Fig. 1) Following PSAX branch PA bifurcation image optimisation, which is typically a rib-space higher than standard, tilt posterior from RPA to “open” LAA and respective pulmonary veins **LAA:** Typically, narrow orifice and posterior (modified PSAX or A2C), finger-like appearance by TTE	
	RAA	Typically, a rib-space higher with medial probe positioning for right atrial appendage. It lies near RSVC insertion **RAA:** typically, triangular with broad based orifice and anterior (modified PSAX), Christa terminalis (prominent muscle bar separating RSVC—RAA) may be seen. Pectinate muscles are course and extend towards the tricuspid valve	
	LSVC	If present, further modified imaging planes (indicator towards 1 o’clock) can visualise the LSVC in its long axis. Typically, a persistent LSVC passes anterior to the LPA before coursing infero-posterior along the left atrioventricular groove into the coronary sinus before draining into the right atrium. In adults, the proximal course of the LSVC is best appreciated from the SSN window	
Modified left PSAX:AoV level (2D & CFD)	**RVOT, PV outflow, MPA & PAs**	**Pulmonic outflow assessment;** –*Inspect and interrogate for obstruction* (*sub-, valve, supra-*)* and/or regurgitation as* *detailed by BSE minimum dataset *[10, 13, 15] Notes: – A rib-space higher may aid branch PA bifurcation optimisation (right pane) – LPA is right sided in PSAX indicator projection – **Large margins of error can exist with respect to RVOT/outflow dimensions and the rib space the probe is positioned within**	
	**Pulmonary veins**	**By TTE, it is generally accepted within CHD that at least 3 pulmonary veins are observed returning to the LA (from any imaging plane)** LAA (bi-directional CFD) & LUPV posterior, when in PSAX window	
		LLPV—red	
		RLPV—red RUPV—blue (often difficult to appreciate in this view) **Note:** Pulmonary veins can be appreciated from numerous windows. It may also be possible in some populations to appreciate pulmonary veins via the SSN “Crab" view	
	Eustachian valve/Chiari network	Eustachian valve (#) can sometimes be seen and may be noted to change inflow profile. It is typically a relatively rigid structure, inserting more caudally (infero-posterior), but can also be mobile and fenestrated. It is different to the Chiari network (= **→**) due to its insertion point, which inserts superior-anterior This is a normal anatomical variant. Possible erroneous pathologic interpretation typically includes mass lesion or Cor triatriatum dexter	
Modified high “ductal view” (2D, CFD, CWD) High left parasternal longitudinal / sagittal plane	**Proximal LPA with isthmus/Desc. aorta in long axis**	**Sweep to assess for PDA. Indicator towards 12/1 o’clock, at least one rib space higher and typically immediately adjacent to the left side of the sternum** Often patient needs to be in an extreme left lateral decubitus position to optimise	
Left PSAX: Basal level (2D & CFD)	**MV/LAVV** TV/RAVV	Qualitative assessment of leaflet morphology, thickness, excursion, regurgitation * = Anterolateral commissure # = Posteromedial commissure **Note:** Xplane or 3D imaging is useful to appreciate atrioventricular anatomy in both SAX and the corresponding LAX projection [11], especially if LAVV/RAVV	
	**Left ventricle**	**Qualitative assessment of radial function/RWMA (n: 6)**	
	**Right ventricle**	**Qualitative assessment of radial function. If RV;** A: Anterior RV wall I: Inferior RV wall L: Lateral RV “free” wall	
Left PSAX: Mid-level (2D & CFD)	**Left ventricle** **Right ventricle** **MV/LAVV** **TV/RAVV**	**Qualitative assessment of radial function/RWMA (n: 6)** **Can assess septal curvature loading in systole (pressure) and diastole (volume).** Can utilise Eccentricity index (just below level of MV leaflets), ensuring correct measurement alignment Normal Eccentric index: < 1.2 **Qualitative assessment of radial function** A: Anterior RV wall I: Inferior RV wall L: Lateral RV “free” wall Assess chordae and papillary morphology/apparatus abnormalities * = Lateral papillary muscle # = Medial papillary muscle	
Left PSAX: Apical level (2D & CFD)	**Left ventricle**	**Qualitative assessment of radial function/RWMA (n: 4)**	
SSN long axis (2D & CFD)	**Aortic arch**	Have good head tilt/shoulders raised. This standard window should be modified to appreciate optimised vessel calibre, including; – Asc. Ao – Transverse Arch (between BCA/IA & LCCA) – Desc. Ao – RPA (enface) – Head and neck vessels (BCA/IA, LCCA, LSA) **Abnormal colour patterns.** Assess for coarctation, PDA or anomalous pulmonary vertical vein **Note:** A right sided aortic arch will require a degree of rightward angulation to appreciate Desc. Ao., and LPA in their respective long axis projections	
SSN long axis (PWD/CWD)	**Aortic arch**	**Individual vessels can be interrogated by Doppler** (including Pedof probe if warranted)**;** – Distal Asc. Ao: CWD – Desc. Ao.: PWD (stepdown if warranted) & CWD **Normal:** Triphasic signal. Some patients may exhibit reduced elastic compliance and loose triphasic profile, but still be considered normal –Head and neck vessels (BCA/IA, LCCA, LSA) If there is evidence of continuous antegrade diastolic forward flow (“diastolic tail”), likely to reflect a degree of narrowing/coarctation of the aorta	
		If holo-diastolic diastolic flow reversal, likely significant aortic regurgitation, but consider; – PDA with significant pulmonary “run-off” – Aortopulmonary window defect – Major aortopulmonary collateral arteries – Unrepaired truncus – Large Blalock-Taussig shunt – Duct dependent flow; i.e. AoV atresia, interrupted aortic arch	
Leftward modified SSN long axis (2D, CFD, PWD, CWD)	**LPA** Innominate vein	Sweep left lateral to open LPA in long axis (LPA is anterior to Desc. Ao.), assess for obstruction, flow reversal and/or use if PDA and if LPA not well seen previously in other imaging windows (typically adult cohorts) Note: In the presence of valvular or supravalvular pulmonary obstruction, mixed/residual flow acceleration is often exhibited in the LPA, which itself may be of normal calibre Often seen in many SSN views but may run retro-aortic as a normal variant	
	LSVC	If present, further modified imaging planes can visualise the LSVC in its long axis. Typically, a persistent LSVC passes anterior to the LPA before coursing infero-posterior along the left atrioventricular groove into the coronary sinus before draining into the right atrium Sometimes bilateral SVCs anatomies will have a bridging vein, that is often difficult to appreciate by TTE	
SSN short axis (2D & CFD)	**Arch sidedness**	Indicator at 3 o’clock. Further rotation of indicator to 4/5 o’clock can help illustrate the BCA/IA in its long axis projection **Sweep superior/anterior and follow first head and neck vessel and follow branching pattern** **Note: First arch vessel is typically BCA/IA and arch sidedness is typically contralateral to its direction** **Normal:** Left sided aortic arch: BCA/IA is rightward and bifurcates	
		**Right sided aortic arch:** BCA/IA is leftward and in normal head and neck vessel arrangement will also bifurcate **Note: Left and right aortic arch refers to which side the bronchus/trachea are crossed by the aortic arch, not which side of the midline the aortic root or Desc. Ao. takes** There are many variations of head and neck vessel arrangement. Of which, TTE is often sub-optimal to provide detailed diagnosis	
SSN short axis (2D & CFD)	RPA Venous anatomy	Assess calibre and flow pattern, if not assessed earlier in study. Optimise Doppler angle. The more perpendicular, the less reliable due to angle of incidence principle Long axis Innominate vein to RSVC **Normal: Innominate vein (AKA brachiocephalic vein) is typically anterior to the Asc. Ao. and drains to the RSVC**	
SSN short axis: “Crab view” (2D & CFD)	Pulmonary veins: LLPV LUPV RLPV RUPV	Pulmonary veins can be appreciated from numerous windows. It is largely accepted that “crab view” in adult populations is often sub-optimal **Normal;** LLPV—red LUPV—blue	
		**Normal;** RLPV—red RUPV—blue	
High right parasternal: Longitudinal view (2D, CFD, PWD, CWD)	Outflow/aortic root	Indicator at ~ 11–1 o’clock. Ascending aortic vessel can be interrogated (Pedof probe is recommended) for obstruction. Often useful in interrogating any valvular/supra-valvular obstruction with eccentric forward flow jets that are often difficult to align optimally elsewhere	
	IAS, RSVC, IVC	Indicator at ~ 12 o’clock Often patient needs to be in the right-sided decubitus position	
Supraclavicular view (2D, CFD, PWD, CWD)	SVC	Indicator at ~ 12 o’clock A right supraclavicular view may be used where necessary (i.e. assessment in Fontan circuit: RSVC Glenn ± distal conduit anastomosis sites). The same interrogation may also be performed in the left supraclavicular view for left SVC anatomy In adult populations, RSVC is not always best appreciated from the SSN SAX imaging, and this window can be adopted

**Table 2:** Abbreviations

A2C: Apical two chamber	PAP: Pulmonary artery pressure
A3C: Apical three chamber	PASP: Pulmonary artery systolic pressure
A4C: Apical four chamber	PDA: Patent ductus arteriosus
A5C: Apical five chamber	PG: Pressure gradient
Ab. Ao.: Abdominal aorta	PHT: Pressures half time
AMVL: Anterior mitral valve leaflet	PLAX: Parasternal long axis
AoV: Aortic valve	PMVSD: Peri-membranous ventricular septal defect
Asc. Ao.: Ascending aorta	PR: Pulmonary regurgitation
ASD: Atrial septal defect	PSAX: Parasternal short axis
AVA: Aortic valve area	PV: Pulmonary valve
BCA: Brachiocephalic artery	PWD: Pulsed wave Doppler
BSA: Body surface area	RA: Right atrium
BSE: British Society of Echocardiography	RAO: Right anterior oblique
CFD: Colour flow Doppler	RAP: Right atrial pressure
CS– Coronary sinus	RAVV: Right atrioventricular valve
CWD: Continuous wave Doppler	RCA: Right coronary artery
Desc. Ao.: Descending aorta	RLPV: Right lower pulmonary vein
DVI: Dimensionless velocity index	RPA: Right pulmonary artery
EROA: Effective regurgitant orifice area	RSVC: Right superior vena cava
IA: Innominate artery	RUPV: Right upper pulmonary vein
IAS: Inter atrial septum	RV: Right ventricle
IVC: Inferior vena cava	RVSP: Right ventricle systolic pressure
IVSDd/s: Inter ventricular septum diameter, diastole/systole	RVIDd: Right ventricle internal diameter, diastole
LA: Left atrium	RVOT: Right ventricle outflow tract
LAA: Left atrial appendage	RWMA: Regional wall motion abnormality
LAVV: Left atrioventricular valve	SC4C: Subcostal four chamber
LCCA: Left common carotid artery	SoV: Sinus of valsalva
LCA: Left coronary artery	SSN: Suprasternal notch
LLPV: Left lower pulmonary vein	ST Jcn: Sinotubular junction
LPA: Left pulmonary artery	TAPSE: Tricuspid annular planar systolic excursion
LSVC: Left superior vena cava	TDI: Tissue Doppler imaging
LUPV: Left upper pulmonary vein	ToF: Tetralogy of Fallot
LV: Left ventricle	TR: Tricuspid regurgitation
LVIDd/s: Left ventricle internal diameter, diastole/systole	TTE: Transthoracic echocardiography
LVOT: Left ventricle outflow tract	TV: Tricuspid valve
LVPWDd/s: Left ventricle posterior wall diameter, diastole/systole	Vmax: Maximum velocity
MAPSE: Mitral annular planar systolic excursion	VSD: Ventricular septal defect
MM: M-mode	VTI: Velocity time integral
MPA: Main pulmonary artery	2D: Two dimensional
MV: Mitral valve	3D: Three dimensional
PA: Pulmonary artery

**Table 3:** Summarised guideline for performing a comprehensive sequential segmental TTE in the CHD patient

View	Modality	Anatomy/image/measurement
Subcostal	2D, CFD	Visceral/inferred atrial situs
	2D, CFD	IVC connection, size and collapsibility
	2D, CFD, PWD	Abdominal aorta
	2D	Cardiac position and apex position
	2D, CFD, PWD	Long axis & bicaval IAS assessment
	2D	AV connection, ventricular morphology
	2D, CFD	VA connection, vessel relationship
	2D, CFD, PWD/CWD	RV inflow/outflow
	2D	Pericardial effusion
Apical: A4C	2D, CFD, PWD	LA size, pulmonary vein assessment
	2D, CFD, PWD/CWD	Left atrioventricular valve appearance, and function
	2D, MM, TDI	Ventricular morphology, size, thickness, and function
Apical: A5C	2D, CFD, PWD/CWD	Outflow assessment
Apical: A2C	2D, CFD, PWD	LA size/pulmonary vein assessment
	2D, CFD, PWD/CWD	Left atrioventricular valve appearance, and function
	2D	Ventricular morphology, size, thickness, and function
Apical: A3C	2D, CFD, PWD	LA size/pulmonary vein assessment
	2D, CFD, PWD/CWD	Left atrioventricular valve appearance, and function
	2D	Ventricular morphology, size, thickness, and function
	2D, CFD, PWD/CWD	Outflow assessment
Apical		
A4C, A2C, A3C	2D, GLS, 3D	Focus systemic ventricle assessment
Apical:	2D	RA size
Mod. right heart	2D, CFD, PWD/CWD	Right atrioventricular valve appearance, and function
	2D, MM, TDI	Ventricular morphology, size, thickness, and function
Parasternal	2D	AV and VA connections
Long axis	2D, CFD, PWD/CWD	Atrioventricular valves’ appearance, and function
	2D, MM	Ventricular morphology, size, thickness, and function
	2D, CFD, PWD/CWD	Outflows’ assessment
	2D, CFD, CWD	Inflows’ assessment
	2D, CFD	VSD sweep
Parasternal	2D	AV and VA connections
Short axis	2D, CFD	Aorta cusp morphology
	2D, CFD	Coronary origins (coronary specific settings)
	2D, CFD, PWD/CWD	Right atrioventricular valve appearance, and function
	2D, CFD, PWD/CWD	Left atrioventricular valve appearance, and function
	2D, CFD, PWD/CWD	RVOT, outflow & branch PA assessment (i.e. PDA)
	2D, CFD, PWD	Pulmonary vein assessment
	2D, CFD, PWD/CWD	Atrial septum assessment
	PSAX Base—2D, CFD	LV size, thickness, and function
	PSAX mid—2D, CFD	LV size, thickness, and function
	PSAX Apical—2D, CFD	LV size, thickness, and function
	2D, CFD	VSD sweep
Ductal view	2D, CFD, CWD	PDA assessment
Suprasternal	2D, CFD, PWD/CWD	Arch calibre, PDA/CoA assessment
Long axis	2D, CFD	Head and neck vessel pattern
	2D, CFD, PWD/CWD	LPA assessment
Suprasternal	2D, CFD	Arch sidedness
Short axis	2D, CFD, PWD/CWD	Branch PA assessment
	2D, CFD, PWD	“Crab view” pulmonary vein assessment
	2D, CFD	RSVC ± LSVC, innominate vein anatomy

**Table 4:** Sample sequential segmental CHD TTE report (normal)

Conclusion:
No cardiac disease identified Structurally normal heart with good biventricular size and function No significant valve abnormalities No ASD/VSD/PDA
Situs solitus, left-sided, leftward apex (levocardia) Concordant AV/VA connections. Normal spatially orientated great arteries No pericardial effusion Atrial septum intact
IVC and RSVC and non-dilated CS drain into non-dilated right atrium (ESA – __cm^2^). IVC normal size > 50% collapse (Est. RAP <5mmHg) Tricuspid valve is thin and mobile, opens well. Normal tricuspid valve inflow. Trivial tricuspid regurgitation Vmax—__mmHg, Est RVSP/PASP – __mmHg + RAP Normal right ventricular structure. Non-dilated, non-hypertrophic right ventricle with good systolic function; RVD1 – __mm, RVD2 – __mm, RVD3 – __mm, TAPSE – __mm, RV S' – __cm/sec, RV EDA – __cm2, RV FAC% – __% No right ventricular outflow tract obstruction. Pulmonary valve is anterior and leftward Pulmonary valve is thin and mobile, opens well. Trivial pulmonary incompetence [if complete PR Doppler, est. mean PAP and PAEDP] Confluent, good sized pulmonary arteries. No PDA
At least 3 pulmonary veins seen returning to non-dilated left atrium (ESV – __ml) Mitral valve is thin and mobile, opens well. Normal mitral valve inflow. Trivial mitral regurgitation Non-dilated, non-hypertrophic left ventricle. Good left ventricular systolic and diastolic function IVSd – __mm, LVPWDd – __mm, LVIDd – __mm, FS% – __%, Teicholz LVEF – __% LV EDV (Biplane) – __ml, LVEF (Biplane) – __% MAPSE – __mm, Lat S' – __cm/sec, Sep S'—__cm/sec E/e’ – Interventricular septum intact. No left ventricular outflow tract obstruction Trileaflet aortic valve that is thin and mobile, opens well. No Aortic regurgitation. Normal origins of coronary arteries Unobstructed, left sided arch. No CoA. Pulsatile Ab. Aorta

## Data Availability

Not applicable.
